# Association of beta_2_-adrenergic receptor gene polymorphisms and nocturnal asthma in Saudi patients

**DOI:** 10.4103/1817-1737.78416

**Published:** 2011

**Authors:** Abdullah M. Al-Rubaish

**Affiliations:** *Department of Internal Medicine, King Fahad Hospital of the University, College of Medicine, University of Dammam, Dammam, Saudi Arabia*

**Keywords:** Asthma, β_2_-adrenergic receptor, frequency, polymorphism, Saudi Arabia

## Abstract

**BACKGROUND AND OBJECTIVES:**

Two polymorphisms of beta_2_-adrenergic receptor (β_2_-AR) gene, namely the substitution from arginine (Arg) to glycine (Gly) at codon 16 and from glutamine (Gln) to glutamic (Glu) at codon 27, are linked with functional changes in the β_2_-AR in the respiratory system even though they are not deemed to be susceptibility genes for asthma *per se*. The objective of this study was to investigate this association in a subset of asthmatic patients, namely those with nocturnal asthma.

**METHODS:**

The β_2_-AR gene polymorphisms at codon 16 and 27 were assessed in 40 patients clinically diagnosed with nocturnal asthma and 96 normal controls. Genomic DNA was obtained from whole blood and genotyping was carried out by a PCR based restriction fragment length polymorphism technique.

**RESULTS:**

There was a statistically significant difference in genotype frequencies at codon 16 (Arg/Gly) between nocturnal asthmatic patients and normal control subjects (*P* < 0.05). However, there was no statistically significant difference in allele frequencies between the two groups. In addition, there was a significant association between Arg16-Gly genotype with nocturnal asthma compared to homozygous Gly16 (codominant model *P* = 0.0033, OR = 3.69: 95% CI: 1.49-9.12). However, there were no statistically significant differences in genotype and allele frequencies at codon 27 (Gln/Glu) between the normal control and nocturnal asthmatic groups (χ^2^ = 1.81, *P* = 0.41). The results also indicate that linkage disequilibrium existed between the β_2_-AR codon 16 and β_2_-AR codon 27 polymorphism (|D´| = 0.577). The data for all haplotypes did not show a statistically significant association.

**CONCLUSION:**

We present the genotype and allele frequencies of β_2_-AR gene polymorphisms in normal Saudi subjects and nocturnal asthmatic patients. There was a significant difference in genotype frequencies at codon 16 (Arg/Gly). However, our study indicates a poor association of individual single nuceotide polymorphisms with nocturnal asthma.

The gene for the human beta_2_-adrenergic receptor (β_2_-AR) is located on chromosome 5q31.[1] A number of single nucleotide polymorphisms (SNPs) in the β_2_-AR gene have been identified.[2–4] The most frequent SNPs are due to two missense mutations, which occur in the coding region of the intronless β_2_-AR gene. The first SNP (A > G) at nucleotide 46 causes the substitution of glycine (Gly) for arginine (Arg) at codon 16. The second SNP (C > G) at nucleotide 79 results in the substitution of glutamic (Glu) acid for glutamine (Gln) at codon 27. It has been reported that there are ethnic variations in the prevalence of these two β_2_-AR SNPs.[5,6] Reports have indicated that these polymorphisms, which cause downregulation of the β_2_-AR, play a significant role in bronchial asthma since they are linked with functional changes in the β_2_-AR in the respiratory system.[6,7] A study by Reihsaus *et al*. and other studies found no difference in allele frequency of these two polymorphisms in patients with and without asthma.[2,8,9]

On the other hand, a number of studies have shown that there is increased risk of asthma susceptibility in patients with these polymorphisms.[10–12] However, a meta-analysis study concluded that neither polymorphism influences the risk for asthma.[13] Subsequent studies have been performed to determine whether these polymorphisms influence more specific asthma phenotypes, such as the severe form of the disease or nocturnal asthma.[13–17] Nocturnal asthma constitutes a subset of asthma, which is associated with significant decline in pulmonary function and increase in airway inflammation at night.[18]

The Eastern Province of Saudi Arabia has one of the highest incidence rates of asthma in the Kingdom.[19,20] A study confirmed that asthma, chronic obstructive pulmonary disease, and pneumonia are the leading causes of hospitalization of patients with respiratory disorders in the Eastern Province.[20] The allele frequencies of these two polymorphic sites have been reported for the normal Saudi population.[5] The only existing report concerning the association of the prevalence of β_2_-AR and asthma indicates that there is no statistically significant association between these polymorphisms and asthma *per se* in the population of the Eastern Province.[21] However, there have been reports concerning the association of these polymorphic sites with nocturnal asthma.[15–17] This prompted us to carry out this study to determine the prevalence of these polymorphic sites in normal Saudi individuals and patients with nocturnal asthma.

## Methods

Forty Saudi patients attending King Fahad Hospital of the University, Al-Khobar, Saudi Arabia over a period of one year between 2009 and 2010, who had been clinically diagnosed with nocturnal asthma were included in this case–control study. The participating patients were well-defined nocturnal asthmatics. It must be noted that this group of patients differs from those included in our previous asthma study. The control group was comprised of 96 subjects recruited from the general population, who were in the same age range. The sole exclusion criterion for control subjects was past, present or family history of asthma. All participants were requested to sign an informed written consent prior to participation in the study. Genomic DNA was obtained from 300 μL whole blood using QIAamp Blood Kit (Qiagen, Hilden, Germany) according to the manufacturer’s protocol. Genotypes of samples were determined using restriction fragment length polymorphism as described in the literature.[13] In brief, a 308 bp region of the β_2_-AR gene spanning both polymorphic sites was amplified. The following primers 5’-CCT TCT TGC TGG CAC CCC AT-3’ (sense) and 5’-GGA AGT CCA AAA CTC GCA CCA-3’ (antisense) were used. The PCR reaction volume of 25 μL contained 100–250 ng of DNA, 1.5 mM MgCl_2_, 200 μM dNTP, and 0.25 U Tag DNA polymerase in a standard PCR buffer. PCR cycles involved an initial 5 min at 94°C followed by 30 cycles at 94°C for 30 s, 58°C for 30 s, 72°C for 45 s, followed by a final extension at 72°C for 10 min. The PCR product was then digested with restriction enzyme *Nco*I for Arg16Gly and *Bbv*I for the Glu27Gln polymorphism at 45°C for 1 h. Restriction products were separated on 3% agarose and visualized under UV illumination following ethidium bromide staining.

Genotype and allele frequencies were estimated by gene counting and expressed as percentages of the total. The χ^2^-test was used to compare differences between groups. A difference was considered statistically significant when *P* < 0.05. Statistical analysis Fisher’s Exact test was used to test whether the frequency distribution of genotypes in the control group was in Hardy–Weinberg equilibrium (HWE). Linkage disequilibrium (LD), haplotypes frequencies estimation, and haplotypes association analysis were performed using the SNPStats web tool for association studies (http://bioinfo.iconcologia.net/SNPstats). The association was expressed as an odds ratio (OR) with the corresponding 95% confidence interval (95% CI).

## Results

A total of 136 samples from 40 patients and 96 age and gender matched controls were analyzed. Table 1 presents the baseline characteristics of the two study groups. The genotype distributions of both loci were in Hardy Weinberg equilibrium in the control group (*P* = 0.057 for β_2_-AR codon 16 and *P* = 0.178 for β_2_-AR codon 27). Table 2 presents the genotype and allele frequencies of β_2_-AR gene polymorphism at codon 16 (Arg 16 Gly) in normal control and nocturnal asthmatic subjects. There was a statistically significant difference in genotype frequencies between patients with nocturnal asthma and normal control subjects (*P* < 0.05). However, there was no statistically significant difference in allele frequencies between the two groups. The data showed that there is a significant association between Arg16-Gly genotype with nocturnal asthma when compared to homozygous Gly16 (codominant model, *P* = 0.0033, OR = 3.69; 95% Cl: 1.49–9.12). Table 3 summarizes the genotype and allele frequencies of β_2_-AR polymorphism at codon 27 (Glu 27 Gln) in normal control and nocturnal asthmatic subjects. No significant statistical differences were found in genotype and allele frequencies between the normal control and nocturnal asthmatic groups (χ^2^= 1.81, *P* > 0.4).

**Table 1:** Baseline characteristics of asthmatic patients and normal control subjects

	Control	Patients
Number	96	40
Age, mean ± SD (years)	12.6 ± 4.2 (range: 5–21 years)	11.4 ± 4.6 (range: 5–20 years)
Gender (males)	60.2%	54.8%
Duration of asthma	None	2–3 years
Severity of symptoms	None	Persistent cough at night
Frequency	None	3–4 times per week
FEV1	Normal	<60%
Treatment	None	Inhaled corticosteroids, salmeterol, and albuterol

**Table 2:** Distribution of genotype and allele frequencies of β-adrenergic receptor at codon 16 (Arg/Gly) in normal and nocturnal asthmatic subjects

Genotype	Homozygous arginine 16	Heterozygous Arg/Gly	Homozygous glycine 16	Total	*P* value
Normal	20 (20.8)	37 (38.6)	39 (40.6)	96	0.004
Nocturnal asthma	4 (10.0)	28 (70.0)	8 (20.0)	40	

**Allele**	**Arginine 16**	**Glycine 16**			

Normal	77 (40.1)	115 (59.9)		192	0.455
Nocturnal asthma	36 (45.0)	44 (55.0)		80	

Figures in parentheses are in percentage

**Table 3:** Distribution of genotype and allele frequencies of β-adrenergic receptor at codon 27 (Glu/Gln) in normal and nocturnal asthmatic subjects

Genotype	Homozygous glutamic 27	Heterozygous Glu/Gln	Homozygous glutamine 27	Total	*P* value
Normal	9 (9.5)	31 (32.6)	55 (57.9)	95	0.405
Nocturnal asthma	7 (17.5)	11 (27.5)	22 (55.0)	40	

**Allele**	**Glutamic**	**Glutamine**			

Normal	49 (25.8)	141 (74.2)		190	0.358
Nocturnal asthma	25 (31.2)	55 (68.8)		80	

Figures in parentheses are in percentage

The results also indicate that linkage disequilibrium existed between the β_2_-AR codon 16 and β_2-_AR codon 27 polymorphism (|D´| = 0.577). The estimated frequencies for various haplotypes in both the control and patient groups and the significance of association with asthma are presented in Table 4. The data for all haplotypes did not show a statistically significant association.

**Table 4:** Comparison of haplotypes frequencies between patients and controls

Haplotype	Patients	Controls	Odds ratio (95% Cl)	*P* value
AG	0.373	0.3661	Reference	
GG	0.3145	0.3795	0.83 (0.42 –1.65)	0.6
GC	0.2355	0.2186	0.99 (0.52 –1.92)	0.99
AC	0.077	0.0359	2.29 (0.48 –10.89)	0.3

## Discussion

Asthma is a complex disease caused by interactions between many genes and environmental factors. Identification of the genetic basis of asthma may contribute to discovery of innovative asthma drugs. Nocturnal asthma represents a subset of asthmatic patients who usually experience worsening symptoms and the obstruction of airflow during the night.[18,22] Nocturnal asthma is a severe form of the disease and it is associated with increased morbidity, which has an extreme negative impact on the quality of life for patients. Patients with nocturnal asthma were generally found to have >15% decrease in lung function during the night.[22] A number of single nucleotide polymorphisms in the β_2_-AR gene namely Arg/Gly at codon 16 and Gln/Glu at codon 27 have been implicated in asthma susceptibility.[10–12] A single amino acid substitution in the structural domains critical for receptor function has been shown to result in significant changes in receptor function.[7] However, these studies have generated conflicting results as to whether these genes alone confer a risk for asthma. Reports have indicated that Gly16 allele is associated with enhanced agonist-mediated downregulation of the receptor, while Glu27 allele enhances resistance to downregulation.[23,24] A meta-analysis study, which was composed of several thousand individuals, suggested that Arg/Gly polymorphism could possibly contribute to nocturnal symptoms in asthmatic patients.[13]

A study of the association of these two polymorphisms and asthma *per se* was recently concluded on a Saudi population residing in the Eastern Province.[21] This study showed that there was a significant difference in genotype frequencies at codon 16 (Arg16Gly) between asthmatic and normal control subjects (*P* < 0.05). However, there was no statistically significant difference in allele frequencies between the two groups (*P* > 0.05). In addition, when comparing the genotype and allele frequencies of Glu27Gln, no significant differences were observed between the asthmatic group and the normal control group.

In this study, we compared the allele frequencies of these two polymorphisms between normal and nocturnal asthmatic patients. There was a statistically significant difference in genotype frequencies at codon 16 (Arg16Gly) between nocturnal asthmatic patients and normal control subjects (*P* < 0.05). However, there was no statistically significant difference in allele frequencies between the two groups (*P* > 0.05). Our results of genotype frequencies of Arg16Gly polymorphism are in line with those of Kaisheng *et al*. who examined the frequency of this polymorphic site of the β_2_-AR gene in nocturnal asthmatic subjects.[15] It is also worth mentioning that there was no statistically significant difference in genotype or allele frequencies between the nocturnal asthmatic patients included in this study and the nocturnal asthmatic patients included in our previous study. When comparing the genotype and allele frequencies of Gln27Glu, no statistically significant differences were observed between the nocturnal asthmatic group and the normal control group (*P* > 0.05).

Previous reports have indicated that the interactions of multiple SNPs within a haplotype influence the phenotype more significantly that individual SNPs.[25] We examined the association of both SNPs and haplotypes with nocturnal asthma and concluded that there was no significant association.

In summary, we present the genotype and allele frequencies of β_2_-AR gene polymorphisms in normal Saudi subjects and nocturnal asthmatic patients. There was a significant difference in genotype frequencies at codon 16 (Arg/Gly). However, our study indicates a poor association of individual single nucleotide polymorphisms with nocturnal asthma. Further studies are required to ascertain the interactions of different haplotypes and the response of patients with different haplotypes to various treatments.
